# Peri-interventional antibiotic prophylaxis in endoscopic valve implantation for lung volume reduction in COPD patients: results from a German multicenter observational cohort

**DOI:** 10.1007/s15010-026-02742-w

**Published:** 2026-02-13

**Authors:** Eva Pappe, Hadis Darvishi, Thomas Sgarbossa, Jacopo Saccomano, Kaid Darwiche, Stefan Andreas, Stephan Eisenmann, Bernd Schmidt, Wolfgang Gesierich, Nicolas Dickgreber, Christian Geltner, Joachim Hans Ficker, Angelique Holland, Björn Schwick, Stephan Eggeling, Ralf Eberhardt, Christian Grah, Christoph Hünermann, Urte Sommerwerck, Andreas Fertl, Sylke Kurz, Peter Schramm, Dinah von Schöning, Leif Erik Sander, Martin Witzenrath, Ralf-Harto Hübner

**Affiliations:** 1 Department of Infectious Disease, Respiratory Medicine and Critical Care, Charité - Universitätsmedizin Berlin, Corporate Member of Freie Universität Berlin and Humboldt- Universität Zu Berlin, Hindenburgdamm 30, 12203 Berlin, Germany; 2 Department of Pulmonology, Lungenklinik Hemer, Hemer, Germany; 3 Center of Respiratory Medicine, Lungenfachklinik Immenhausen, Immenhausen, Germany; 4 Department of Respiratory Medicine, Universitätsklinikum Halle, Halle, Germany; 5 Department of Internal Medicine - Respiratory and Sleep Medicine, DRK Kliniken Berlin Mitte, Berlin, Germany; 6 Department of Respiratory Medicine, Asklepios Lungenklinik Gauting, Gautingen, Germany; 7 Department of Internal Medicine 3 – Pneumology, Klinikum Ibbenbüren, Ibbenbüren, Germany; 8 Pneumology and Internal Medicine, Donauisar Klinikum Landau, Landau an Der Isar, Germany; 9 Department of Internal Medicine 3/Respiratory Medicine, Klinikum Nuernberg, Paracelsus Medical University, Nuernberg, Germany; 10 Department of Respiratory Medicine, Universitätsklinikum Marburg, Marburg, Germany; 11 Department of Respiratory Medicine, Luisenhospital Aachen, Aachen, Germany; 12 Department of Thoracic Surgery, Vivantes-Klinikum Neukölln, Berlin, Germany; 13 Pneumology and Critical Care Medicine, Asklepios Klinik Barmbek, Hamburg, Germany; 14 Department of Respiratory Medicine, Klinik Havelhöhe Berlin, Berlin, Germany; 15 Pneumology, Krankenhaus St. Raphael Niels-Stensen-Kliniken, Ostercappeln, Germany; 16 Pneumology, Krankenhaus Der Augustinerinnen, Cologne, Germany; 17 Pneumology, Krankenhaus Martha-Maria, Munich, Germany; 18 Department of Pulmonology, Evangelische Lungenklinik Berlin, Berlin, Germany; 19 Department of Pulmonology and Pulmonary Oncology, München Klinik Bogenhausen, Munich, Germany; 20 Institute for Medical Microbiology and Virology, University Hospital Leipzig, Leipzig, Germany; 21 German Center for Lung Research (DZL), Berlin, Germany; 22 CAPNETZ Stiftung, Hannover, Germany

**Keywords:** Endoscopic lung volume reduction, Antibiotic prophylaxis, Chronic obstructive pulmonary disease, Procedure-related complications

## Abstract

**Background:**

Endoscopic lung volume reduction (ELVR) using endobronchial valves is an established treatment for advanced COPD and emphysema. To reduce procedure-related complications such as pneumonia or exacerbations, peri-interventional antibiotic prophylaxis is commonly used; however, its clinical benefit remains uncertain. We aimed to evaluate the effect of different peri-interventional antibiotic strategies in a German COPD cohort.

**Methods:**

Comparative analyses were performed using data from 900 patients enrolled in the multicentre, observational German Lung Emphysema Registry (LE-Registry). Patients were categorized by peri-interventional antibiotic strategy: single-dose prophylaxis, prolonged prophylaxis for 5–7 days, or no prophylaxis. Baseline characteristics, airway colonization, lung function, symptom burden, exercise capacity, and adverse events were assessed up to three months after ELVR.

**Results:**

Among 900 patients undergoing ELVR, 104 received single-dose prophylaxis, 344 prolonged 5–7-day prophylaxis, and 309 no antibiotic prophylaxis. Clinical improvements in lung function, symptom burden, and exercise capacity over three months were similar across all groups. Exacerbations occurred in 11.5% of patients receiving single-dose prophylaxis (12/104), 7.6% with prolonged prophylaxis (26/344), and 5.5% without prophylaxis (17/309; *p* = 0.12). Pneumonia was observed in 7.7% (8/104), 3.2% (11/344), and 3.2% (10/309), respectively (*p* = 0.087). Antibiotic-related adverse events were infrequent and mild, occurring only in the prolonged prophylaxis group.

**Conclusion:**

Peri-interventional antibiotic prophylaxis, whether single-dose or prolonged, did not improve clinical outcomes or reduce complication rates following ELVR. These findings indicate limited clinical benefit and support a more targeted, indication-based use of antibiotics in this setting.

**Supplementary Information:**

The online version contains supplementary material available at 10.1007/s15010-026-02742-w.

## Introduction

Endoscopic lung volume reduction (ELVR) with endobronchial valves (EBV) is an established treatment option for patients with severe chronic obstructive pulmonary disease (COPD) suffering from advanced emphysema, whereby one-way valves are bronchoscopically implanted to induce atelectasis of the most diseased lung regions [1]. Treatment with ELVR has shown significant and clinically relevant improvements in lung function, exercise capacity, physical activity, dyspnea severity, and quality of life in COPD patients [2–7].

In addition to appropriate patient selection, the management of procedure-related adverse events is essential. Among the most relevant complications following ELVR are acute exacerbations of COPD (AECOPD) and infections such as pneumonia, both ultimately requiring early valve removal [8]. To mitigate these risks, periprophylactic antibiotic therapy is frequently administered during ELVR and has become routine practice in many centers [1]. Based on early clinical practice and protocols used in landmark trials such as the VENT study, peri-interventional antibiotic prophylaxis has been commonly applied in the context of ELVR. In the VENT trial, antibiotics were administered intravenously immediately before the procedure and for up to 24 h thereafter, followed by an oral course for up to seven days, most frequently using second- or third-generation cephalosporins or fluoroquinolones [7]. However, subsequent ELVR trials have reported peri-interventional antibiotic use inconsistently. In the IMPACT trial, antibiotics were administered for 5–7 days after the intervention, but specific agents were not clearly defined in the main publication [4]. In contrast, the study by *Klooster *et al*.* does not report routine antibiotic prophylaxis [5], and while prophylactic antibiotics are documented in the REACH trial, neither the antibiotic classes nor the duration of treatment are specified [9]. Peri-interventional antibiotic prophylaxis is often considered in patients with severe COPD, who may be at increased risk for post-procedural complications due to impaired lung function and a higher prevalence of chronic airway colonization with pathogenic bacteria [10]. However, to date, there is no conclusive evidence that prophylactic antibiotic therapy provides a clinical benefit in the context of ELVR. Consistent with this uncertainty, the current German guideline on perioperative and peri-interventional antibiotic prophylaxis does not recommend routine antibiotic use for bronchoscopic procedures [11].

Nevertheless, peri-interventional antibiotic prophylaxis is widely used at many German ELVR centers, although clear evidence for its clinical benefit is currently lacking. To date, no study has systematically evaluated the impact of different prophylactic strategies in this setting.

Given the limited evidence, we aimed to determine whether peri-interventional antibiotic therapy administered either as a single dose or as a prolonged 5–7-day regimen reduces complications and improves short-term outcomes compared with no prophylaxis in patients with severe COPD undergoing ELVR within the LE-Registry.

## Methods

### Study design

**Fig. 1:**
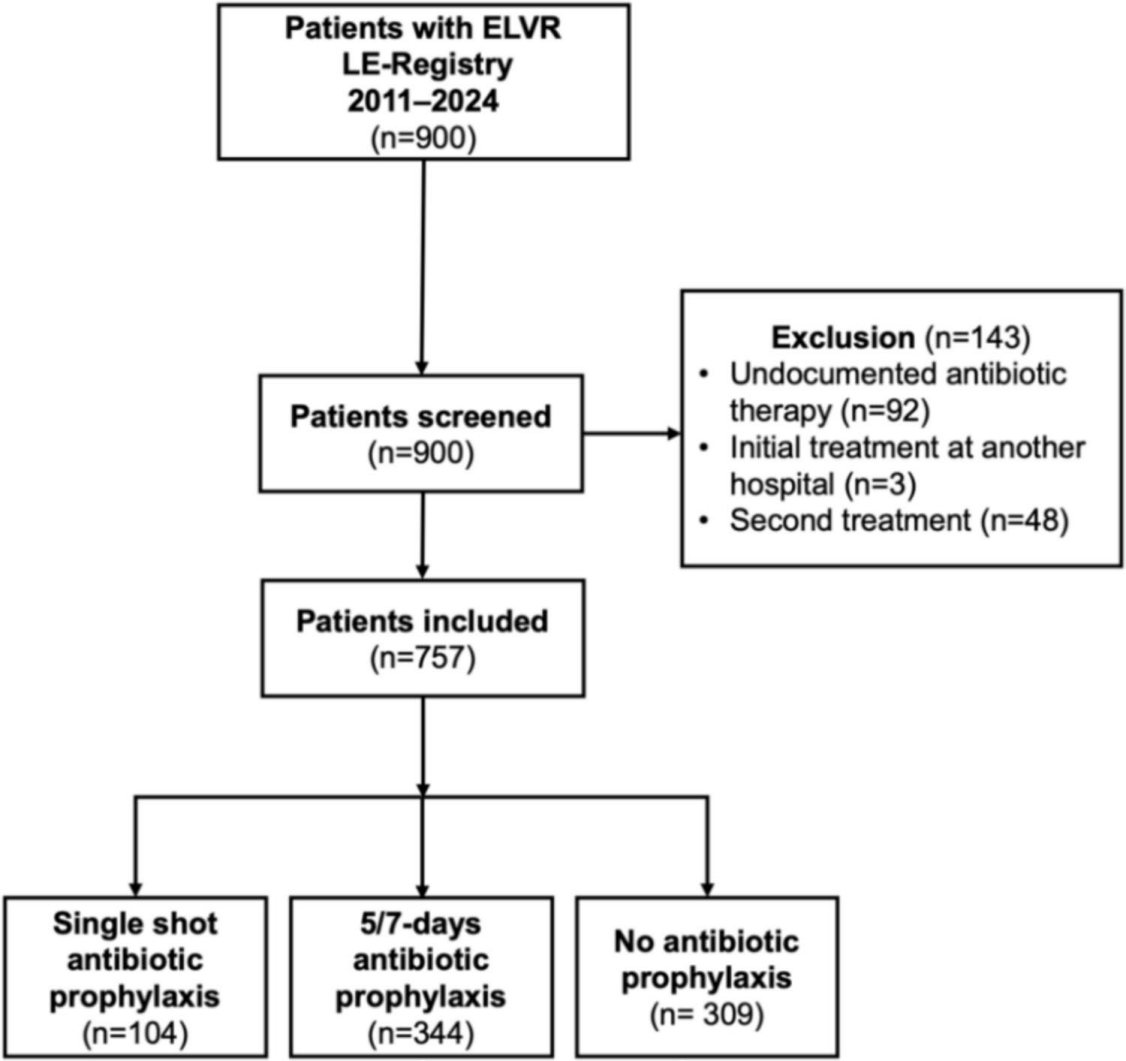
Patient flow diagram Abbreviations: ELVR = Endoscopic lung volume reduction; LE-Registry = German Lung Emphysema Registry

We included patients treated and followed within the LE-Registry (www.lungenemphysemregister.de) between 2011 and 2024 (Fig. 1). The LE-Registry is an open-label, multicenter, observational cohort of COPD patients with severe emphysema undergoing lung volume reduction. Its primary aim is to evaluate outcomes across different techniques within an independently operated, non–industry-funded registry, ensuring assessments are free from external influence.

Data were collected and managed using REDCap [12] hosted at Charité – Universitätsmedizin Berlin. Inclusion and exclusion criteria have been published previously [8, 13–18]. For this study, only patients who underwent ELVR with one-way valves were included. Demographics, lung function, symptom scores, 6-min walk test (6MWD), clinical features, and comorbidities were recorded. Study outcomes were predefined and assessed during the index hospitalization and at 3-month follow-up. Functional outcomes were defined as the change (Δ) between baseline and 3 months in lung function parameters, exercise capacity assessed by 6MWD, pCO₂, and clinical symptom scores, including the COPD Assessment Test (CAT), modified Medical Research Council (mMRC) dyspnea scale, and St George’s Respiratory Questionnaire (SGRQ). Length of hospital stay was assessed as an additional outcome and defined as the number of days from the ELVR procedure to hospital discharge. Furthermore, all peri- and post-interventional adverse events occurred within 3 months after ELVR were recorded, including pneumonia and acute exacerbations. Microbiological samples were obtained as part of routine clinical care according to local practice. Available respiratory samples, including bronchial aspirates and bronchoalveolar lavage fluid collected shortly before or during the ELVR procedure, were analyzed. Microbiological analyses were performed using standard culture-based methods at the local microbiology laboratory.

Patients were grouped according to the peri-interventional antibiotic strategy received: no antibiotics, single-dose prophylaxis (administered during the procedure), or prolonged prophylaxis for 5–7 days. The choice of antibiotic regimen was not standardized across the registry and followed local centre-specific protocols and routine clinical practice, as determined by the treating physician. For Charité patients, missing registry information was supplemented by medical record review. Exclusion criteria were ELVR performed externally, other types of volume reduction, second ELVR-treatment, or missing documentation of antibiotic prophylaxis.

### Ethics approval and consent to participate

The study was approved by the Ethics Committee of Charité – Universitätsmedizin Berlin (EA2/149/17 and EA4/024/22). All patients provided written informed consent. Identifiable information was anonymized, and the study adhered to the Declaration of Helsinki.

### Microbiological samples

Only bronchial washing fluids obtained during flexible bronchoscopy prior to ELVR were analyzed. Samples were collected before EBV implantation and submitted for culture according to local standards. Results were interpreted using the Microbiological-Infectious Quality Standards (MIQ) 07–08: *Infections of the Lower Respiratory Tract, Part I and Part II, 2010* [19].

### Statistical analysis

Analysis was performed using IBM SPSS Statistics (Version 27.0; IBM Corp., Armonk, NY, USA). Continuous variables were tested for normality using the Shapiro–Wilk test and are reported as mean ± standard deviation or median with range or 95% CI, as appropriate; categorical variables are presented as counts and percentages. Changes from baseline to the 3-month follow-up were calculated as absolute deltas (Δ), including changes in forced expiratory volume in 1 s (FEV₁; % predicted and liters), residual volume (RV; % predicted and liters), diffusing capacity of the lung for carbon monoxide (DLCO; % predicted and mmol.min^-1^.kPA^-1^), partial pressure of carbon dioxide (pCO₂; mmHg), 6MWD, CAT, modified MsmMRC, SGRQ. Group differences were assessed using one-way ANOVA for continuous variables and χ^2^ or Fisher’s exact tests for categorical variables. Baseline airway colonization before ELVR was summarized descriptively. All statistical tests were two-sided, with *p* < 0.05 considered significant.

## Results

Figure 1 summarizes ELVR patient cohorts from the LE-Registry, focusing on periinterventional antibiotic prophylaxis. A total of 900 patients were screened. After excluding 143 patients due to undocumented antibiotic therapy (n = 92), initial treatment at another hospital (n = 3), or second ELVR treatment (n = 48), 757 patients were included in the final analysis. Of these, 104 patients received single-shot prophylaxis, 344 received prolonged prophylaxis (5–7 days), and 309 patients received no antibiotic prophylaxis. The most commonly used antibiotic in both prophylaxis groups was ampicillin/sulbactam (see Table 1S in the supplements).

**Table 1:** Baseline characteristics of patients with ELVR (All data presented as mean ± SD/or 95% CI, unless stated otherwise; superscript letters a and b indicate significance

	Single shot antibiotic prophylaxis	Prolonged (5/7-days) antibiotic prophylaxis	No antibiotic prophylaxis	*p*-value
Patients n	104	344	309	
Age years	65.6 ± 6.5	65.5 ± 7.7	66.6 ± 7.8	0.20
Sex n (%)				0.20
Male	57 (54.8)	190 (55.9)	150 (49.0)	
Female	47 (45.2)	150 (44.1)	156 (51.0)	
Comorbidities n (%)				
α_1_-Antitrypsin deficiency	3 (3.0)	21 (6.2)	13 (4.3)	0.40
Cardiovascular disease	19 (18.3)	49 (14.4)	50 (16.3)	0.59
Pulmonary hypertension	12_a_ (11.5)	10_b_ (2.9)	14_b_ (4.6)	**0.001**
Atrial fibrillation	5 (4.8)	20 (5.9)	16 (5.2)	0.89
Arterial hypertension	51 (49.0)	148 (43.4)	140 (45.6)	0.58
Osteoporosis	15 (14.4)	41 (12.0)	31 (10.1)	0.46
Diabetes mellitus type II	9 (8.7)	21 (6.2)	20 (6.5)	0.67
Previous history of lung cancer	1 (1.0)	2 (0.6)	2 (0.7)	0.92
Lung cancer	0 (0)	3 (0.9)	2 (0.7)	0.63
Lung function at baseline				
FEV_1_% of pred	27.7_a_ [26.3;29.1]	29.4_b_ [28.4;30.3]	30.7_b_ [29.6;31.7]	**0.02**
FEV_1_ (L)	0.8 [0.7;0.9]	0.9 [0.7;1.2]	0.8 [0.7;0.8]	0.18
RV % of pred	223.5_a_ [213.0;234.0]	248.82_b_ [242.2;255.4]	249.1.0_b_ [242.6;255.6]	** < 0.001**
RV (L)	5.0_a_ [4.8;5.3]	6.6_b_ [4.7;8.6]	5.6_b_ [5.5;5.8]	** < 0.001**
DLCO % of pred	30.5_a_ [27.8;33.2]	29.1_a_ [27.5;30.8]	32.3_b_ [30.5;34.1]	**0.03**
DLCO mmol.min^−1^.kPa^−1^	2.6 [2.3;2.8]	2.7 [2.2;3.2]	2.5 [2.3;2.6]	0.65
pCO_2_ mmHg	38.7_a_ [37.4;40.0]	42.0_b_ [37.4;40.0]	41.8_b_ [40.8;42.8]	**0.001**
6-MWD m	227.3 [204.9;249.7]	257.9 [246.3;269.5]	257.6 [244.8;270.5]	0.06
CAT points	25.7_a_ [24.3;27.0]	24.0_b_ [23.3;24.8]	25.6_b_ [24.8;26.3]	**0.006**
mMRC points	3.1 [2.9;3.3]	3.0 [2.9;3.1]	3.1 [3.0;3.2]	0.46
SGRQ points	67.3_a_ [64.7;69.8]	65.9_b_ [64.6;67.1]	67.6_a_ [65.4;69.7]	**0.03**

Abbreviations: FEV₁ = Forced expiratory volume in 1 s; RV = Residual volume; DLCO = Diffusing capacity of the lung for carbon monoxide; pCO₂ = Partial pressure of carbon dioxide; 6-MWD = 6-min walking distance; CAT = COPD Assessment Test; mMRC = modified Medical Research Council Dyspnea Scale; SGRQ = St. George’s Respiratory Questionnaire.

Bold values indicate statistically significant differences between groups (*p* 0.05)

### Baseline characteristics

Baseline characteristics of patients undergoing ELVR within the LE-Registry are summarized in Table 1. Age, sex, and most comorbidities were comparable across groups. Pulmonary hypertension was significantly more frequent in the single-shot group (11.5%) than in the prolonged (2.9%) and no-prophylaxis groups (4.6%) (*p* = 0.001). Patients in the single-shot group had the lowest FEV₁% predicted, lower RV, and slightly reduced DLCO (*p* < 0.001; *p* = 0.029). Baseline pCO₂ levels were also lower in this group. CAT and SGRQ scores suggested slightly better symptom control and quality of life in the prolonged prophylaxis group, whereas no significant differences were observed for mMRC or 6-MWD.

### Clinical outcomes

Clinical outcomes at three months were comparable across all groups receiving different peri-interventional antibiotic strategies (Table 2). All three groups showed similar improvements in lung function, exercise capacity, and symptom burden, regardless of the antibiotic regimen applied. Length of hospital stay after ELVR was also comparable.

**Table 2:** Clinical Outcomes after 3-month follow-up based on type of peri-interventional antibiotic therapy Changes over time were assessed by Δ between baseline and 3-month follow-up. (All data presented as mean ± SD/or 95% CI, unless stated otherwise. * Missing values)

	Single shot antibiotic prophylaxis (n = 104)	*	Prolonged (5/7-days) antibiotic prophylaxis (n = 344)	*	No antibiotic prophylaxis (n = 309)	*	*p*-value
Δ FEV1% of pred	4.8 [2.4;7.4]	44	4.2 [3.0;5.4]	15	4.5 [3.4;5.7]	126	0.97
Δ FEV1 (L)	0.1 [0.02;0.2]	43	0.1 [0.8;2]	175	0.1 [0.08;0.2]	117	0.98
Δ RV % of pred	− 24.9 [− 39.5; − 10.3]	44	− 31.6 [− 39.4; − 23.8]	158	− 32.8 [− 42.7; − 23.0]	128	0.89
Δ RV (L)	− 0.5 [− 0.8; − 0.7]	43	− 0.7 [− 0.9; − 0.5]	175	− 0.7 [− 0.9; − 0.5]	120	0.78
Δ DLCO % of pred	2.9 [− 0.6;6.4]	52	1.4 [− 0.4;3.1]	202	2.2 [0.4;4.0]	186	0.64
Δ DLCO (mmol.min − 1.kPa − 1)	0.3 [0.0;0.7]	52	0.1 [− 0.1;0.2]	221	0.2 [0.1;0.4]	192	0.30
Δ pCO2 (mmHg)	− 0.9 [− 2.0; − 0.2]	49	− 1.9 [− 2.9; − 0.9]	169	− 1.7 [− 2.9; − 0.6]	142	0.56
Δ 6MWD m	8.6 [− 12.3;30.1]	54	37.5 [22.8;52.2]	200	35.5 [20.0;51.0]	186	0.11
Δ CAT points	− 3.1 [− 4.8; − 1.5]	48	− 3.0 [− 4.1; − 2.0]	203	− 3.3 [− 4.4; − 2.2]	174	0.90
Δ mMRC points	− 0.6 [− 0.9; − 0.2]	71	− 0.7 [− 0.9; − 0.5]	200	− 0.5 [− 0.7; − 0.3]	195	0.15
Δ SGRQ points	− 10.2 [− 15.4; − 5.0]	65	− 8.6 [− 12.0; − 5.2]	260	− 10.0 [− 13.0; − 6.8]	220	0.78
Duration of hospitalization in days	6.32 ± 4.37		6.66 ± 4.06		6.08 ± 6.32		0.29

Abbreviations: FEV₁ = Forced expiratory volume in 1 s; RV = Residual volume; DLCO = Diffusing capacity of the lung for carbon monoxide; pCO₂ = Partial pressure of carbon dioxide; 6-MWD = 6-min walking distance; CAT = COPD Assessment Test; mMRC = modified Medical Research Council Dyspnea Scale; SGRQ = St. George’s Respiratory Questionnaire.

### Microbiological characterization of lower airway colonization before ELVR

Across all groups, the most frequently detected pathogens in pre-interventional cultures were Escherichia coli (12.0% in the single-shot group, 8.8% in the prolonged group, and 14.3% in the no-prophylaxis group) and Staphylococcus aureus (13.3%, 9.4%, and 3.6%, respectively) (Table 3). Other commonly isolated organisms included Haemophilus influenzae (5.3% in the single-shot group, 7.0% in the prolonged group, none in the no-prophylaxis group), Klebsiella pneumoniae (6.7%, 4.7%, and 3.6%), and Pseudomonas aeruginosa (2.7%, 4.7%, and none). Streptococcus pneumoniae and β-hemolytic streptococci were found in up to 7.1% of patients, while Moraxella catarrhalis, Stenotrophomonas maltophilia, and Aspergillus fumigatus were rarely detected.

**Table 3:** Microbiological characterization of lower airway colonization before endoscopic lung volume reduction in patients treated with endobronchial valves *(ß-haemolytic streptococci including S. pyogenes, S. agalactiae, S. dysgalactiae)*

Pathogens n (%)	Single shot antibiotic prophylaxis	Prolonged (5/7-days) antibiotic prophylaxis	No antibiotic prophylaxis
Staphylococcus aureus	10 (13.3%)	16 (9.4%)	1 (3.6%)
Streptococcus pneumoniae	1 (1.3%)	5 (2.9%)	2 (7.1%)
β-hemolytic streptococci	3 (5.3%)	3 (1.8%)	2 (7.1%)
Haemophilus influenzae	4 (5.3%)	12 (7.0%)	0 (0.0%)
Moraxella catarrhalis	0 (0.0%)	2 (1.2%)	1 (3.6%)
Klebsiella pneumoniae	5 (6.7%)	8 (4.7%)	1 (3.6%)
Escherichia coli	9 (12.0%)	15 (8.8%)	4 (14.3%)
Pseudomonas aeruginosa	2 (2.7%)	8 (4.7%)	0 (0.0%)
Stenotrophomonas maltophilia	0 (0.0%)	3 (1.8%)	0 (0.0%)
Aspergillus fumigatus	1 (1.3%)	0 (0.0%)	1 (3.6%)

### Adverse events

Adverse events within three months after ELVR are summarized in Table 4. The incidence of pneumothorax, exacerbations, and pneumonia did not differ significantly between groups. Exacerbation and pneumonia rates were numerically highest in the single-shot group and lowest in the no-prophylaxis group, although these differences did not reach statistical significance (*p* = 0.12 and *p* = 0.09, respectively). Severe complications such as sepsis, hemoptysis, ICU admission, mechanical ventilation, and death were rare and occurred at similarly low frequencies across all groups.

**Table 4:** Adverse events 3 months after endoscopic lung volume reduction in patients treated with endobronchial valves dependent on peri-interventional antibiotic therapy

	Single shot antibiotic prophylaxis	Prolonged (5/7-days) antibiotic prophylaxis	No antibiotic prophylaxis	*p*-value
Pneumothorax n (%)	19 (9.6)	55 (16.0)	41 (13.3)	0.23
Exacerbations n (%)	12 (11.5)	26 (7.6)	17 (5.5)	0.12
Pneumonia n (%)	8 (7.7)	11 (3.2)	10 (3.2)	0.09
Sepsis n (%)	1 (1.0)	0 (0.0)	0 (0.0)	n.a
Hemoptysis n (%)	1 (1.0)	6 (1.7)	1 (0.3)	n.a
ICU n (%)	3 (2.9)	8 (2.3)	7 (2.3)	n.a
Mechanical ventilation n (%)	2 (1.9)	0 (0.0)	2 (0.7)	n.a
Death n (%)	1 (1.0)	1 (0.3)	0 (0.0)	n.a

Abbreviations: ICU = Intensive care unit.

### Antibiotic-associated complications

Antibiotic-associated complications were rare across all groups and are summarized in Table 5. No cases of anaphylaxis were reported. Minor adverse events, such as skin rash (0.3%) and diarrhea (0.6%), occurred exclusively in the prolonged prophylaxis group. No such events were observed in the single-shot group.

**Table 5:** Antibiotics-associated adverse events after endoscopic lung volume reduction in patients treated with endobronchial valves at Charité-Universitätsmedizin, Berlin (*Vaginal mycosis)

	Single shot antibiotic prophylaxis		Prolonged (5/7-days) antibiotic prophylaxis		No antibiotic prophylaxis		*p*-value
Skin rash n (%)	0	(0.0)	1	(0.3)	0	(0.0)	n.a
Anaphylaxis n (%)	0	(0.0)	0	(0.0)	0	(0.0)	n.a
Diarrhea n (%)	0	(0.0)	2	(0.6)	0	(0.0)	n.a
Other* n (%)	0	(0.0)	1	(0.3)	0	(0.0)	n.a

## Discussion

Peri-interventional antibiotic prophylaxis, whether as a single shot or over a prolonged period, did not significantly reduce complication rates after ELVR. Clinical outcomes, including lung function, symptom burden, and quality of life, were comparable across groups. Pneumonia and AECOPD incidence was low in patients without prophylaxis but comparatively higher for pneumonia in those receiving a single-shot regimen, while prolonged prophylaxis showed similar rates to no prophylaxis. Antibiotic-related side effects and airway colonization occurred infrequently and without relevant differences.

ELVR is a less invasive alternative to surgical lung volume reduction for advanced emphysema [1]. Randomized trials reported FEV₁ improvements of 100–150 mL and RV reductions of 400–700 mL within 3–6 months [2–7, 20, 21]. In our study, median improvement after 3 months was + 80 mL in FEV₁ and –640 mL RV, independent of antibiotic prophylaxis, suggesting that infection control is not the main driver of functional benefit. There are no standardized recommendations on peri-interventional antibiotics in ELVR. In the first pilot study for ELVR, the VENT trial, prophylactic antibiotics were administered shortly before and up to 7 days after valve implantation [7]. The rationale for prolonged antibiotic therapy is based on the assumption that immobilization and the presence of foreign material may promote secretion retention and microbial colonization. Indeed, biofilm formation has been demonstrated on explanted endobronchial valves, supporting the concept that microbial communities can persist on device surfaces [22]. Subsequent large, randomized ELVR studies employed different prophylactic regimens. For instance, the IMPACT study used prolonged antibiotic therapy for 5 to 7 days [4], whereas the REACH trial administered only a single-dose antibiotic therapy during the bronchoscopy procedure [9]. Despite these varied approaches, no systematic investigation has been conducted to determine the effect of prophylactic antibiotic administration during ELVR. To our knowledge, our study is the first to evaluate whether different regimens of prophylactic antibiotics impact the outcomes after ELVR.

Despite its minimally invasive nature, ELVR is associated with several potential complications that require careful consideration and management. One of the potential adverse events are exacerbations and pneumonia following EBV implantation, which can lead to EBV removal [8]. In our study, adverse event rates within 3 months ranged from 3.2–7.7% for pneumonia and 5.5–11.5% for AECOPD and did not differ significantly between groups. Although the absolute number of pneumonia and AECOPD events was higher in the single-dose group, this cohort also exhibited more advanced disease at baseline, suggesting that these differences are more likely related to baseline severity rather than the antibiotic strategy itself. Overall, no clear association was observed between the use or duration of peri-interventional antibiotic prophylaxis and the occurrence of adverse events following ELVR. Other reports have suggested benefit: Abia-Trujillo et al. found fewer exacerbations with prophylaxis [23], but their non-prophylaxis group was very small (n = 26) compared with ours (n = 309). Our larger data set therefore provides a more robust estimate of the effect of peri-interventional antibiotics.

Chronic airway colonization is common in ELVR patients [10]. Long-term follow-up after EBV implantation showed increased prevalence of Staphylococcus aureus, Pseudomonas aeruginosa, Stenotrophomonas maltophilia, Moraxella catarrhalis, and anaerobes [24]. However, colonization was not directly associated with poorer function. Distinguishing colonization from infection remains challenging, and outcomes are influenced by host immunity and comorbidity burden.

This study has several limitations. The observational design precludes causal inference, and the absence of randomization may result in residual confounding. In particular, the choice of peri-interventional antibiotic regimen was not standardized across the registry but followed local centre-specific protocols and routine clinical practice. Consequently, centre-related characteristics, including procedural experience and peri-interventional management routines such as patient preparation, mobilization protocols, inhalation therapy, and physiotherapy, may have influenced both treatment allocation and clinical outcomes independently of antibiotic use.

Furthermore, registry-based data may incompletely capture comorbidities, baseline parameters, or adverse events. Follow-up documentation was less complete than baseline, with a substantial proportion of missing follow-up data, reflecting the inherent characteristics of registry-based data collection and a well-recognized limitation of previous studies based on the LE-Registry [13–15]. This limited the comprehensive assessment of outcomes at later time points and may have introduced bias. In particular, incomplete follow-up may have resulted in underreporting of adverse events. Event rates for pneumonia and AECOPD were low, and some exacerbations were only captured when hospital treatment was required. Owing to missing data and the limited number of outcome events, robust multivariable regression or time-to-event analyses were not feasible.

Despite these limitations, this study has notable strengths. It represents the largest multicenter cohort to date evaluating peri-interventional antibiotic strategies in ELVR. The LE-Registry includes more than 700 well-characterized patients and provides comprehensive real-world data, enabling a systematic description of clinical outcomes and adverse events across different centres and routine clinical practices.

Although residual confounding cannot be ruled out, particularly in light of the observational design and the substantial proportion of missing follow-up data, our results indicate that peri-interventional antibiotic prophylaxis, whether administered as a single dose or as prolonged therapy, was not associated with measurable improvements in clinical outcomes or reductions in adverse events after ELVR in routine clinical practice.

In line with principles of antimicrobial stewardship, the routine use of prophylactic antibiotics in ELVR should therefore be critically reconsidered, as no clear benefit was observed while potential risks such as adverse effects and antimicrobial resistance remain. Antibiotic therapy may still be appropriate in selected patients, for example in cases of suspected post-interventional infection after EBV implantation or in patients with chronic airway colonization, where a targeted short-term course or long-term modulatory treatment (e.g. azithromycin) may be clinically justified. Such approaches should remain individualized rather than applied routinely.

To strengthen the evidence base, randomized controlled trials with larger cohorts and extended follow-up are needed. Future studies should better characterize exacerbations and infectious events, examine microbiological dynamics such as valve colonization and biofilm formation, and integrate measures of symptom burden and patient-reported outcomes.

This multicenter registry analysis provides real-world evidence on peri-interventional antibiotic strategies in ELVR and indicates no measurable benefit of routine prophylactic antibiotic use with respect to clinical outcomes or adverse events. Improvements in lung function, exercise capacity, and quality of life were observed across all treatment groups, and pneumonia and exacerbations remained infrequent. While the observational design and incomplete follow-up warrant cautious interpretation, these findings support a more differentiated, individualized use of antibiotics rather than routine prophylaxis in ELVR.

## Supplementary Information

Below is the link to the electronic supplementary material.

Supplementary file1 (DOCX 20 KB)

## Data Availability

Data can be made available on request to the corresponding author.

## Abbreviations

AECOPD: Acute exacerbation of chronic obstructive pulmonary disease
CAT: COPD assessment test
COPD: Chronic obstructive pulmonary disease
DLCO: Diffusing capacity of the lung for carbon monoxide
EBV: Endobronchial valve
ELVR: Endoscopic lung volume reduction
FEV₁: Forced expiratory volume in 1 s
ICU: Intensive care unit
LE-Registry: German lung emphysema registry
MIQ: Microbiological-infectious quality standards
mMRC: Modified medical research council dyspnea scale
RV: Residual volume
SGRQ: St. George’s respiratory questionnaire
6MWD: Six-minute walking distance
