# Supplementary material for: Peri-interventional antibiotic prophylaxis in endoscopic valve implantation for lung volume reduction in COPD patients: results from a German multicenter observational cohort
Source: Infection. 2026 Feb 13;54(3):1279–88. doi: 10.1007/s15010-026-02742-w (PMC13323262; doi:10.1007/s15010-026-02742-w)
Supplement: Supplementary file 1 — Supplementary file1 (DOCX 20 KB) [file 15010_2026_2742_MOESM1_ESM.docx]

**Supplement material**

**Supplement table 1: Chosen antibiotic treatment of patients treated with endobronchial valves dependent on peri-interventional antibiotic therapy regime**

| **Antibiotic given n (%)** | **Single shot antibiotic prophylaxis (n=104)** | | **Prolonged (5/7-days) antibiotic prophylaxis (n=344)** | |
| --- | --- | --- | --- | --- |
| Ampicillin/Sulbactam | 90 | (86.54) | 155 | (45.06) |
| Amoxicillin/Clavulansäure | 4 | (3.85) | 104 | (30.23) |
| Azithromycin | 0 | (0.00) | 24 | (6.98) |
| Piperacillin/Tazobactam | 1 | (0.96) | 15 | (4.36) |
| Ceftriaxon | 1 | (0.96) | 11 | (3.20) |
| Levofloxacin | 6 | (5.77) | 10 | (2.91) |
| Moxifloxacin | 0 | (0.00) | 6 | (1.74) |
| Ciprofloxacin | 0 | (0.00) | 4 | (1.16) |
| Meropenem | 1 | (0.96) | 2 | (0.58) |
| Ampicillin | 0 | (0.00) | 1 | (0.29) |
| Clarithromycin | 0 | (0.00) | 1 | (0.29) |
| Cefazolin | 0 | (0.00) | 1 | (0.29) |
| Cotrimoxazol | 0 | (0.00) | 1 | (0.29) |
| Combination | 1 | (0.96) | 5 | (1.45) |
| Unknown | 0 | (0.00) | 3 | (0.87) |
